# The influence of concentration of micro-rna hsa-mir-370-3p and CYP2D6*4 on equilibrium concentration of mirtazapine in patients with major depressive disorder

**DOI:** 10.1192/j.eurpsy.2021.1214

**Published:** 2021-08-13

**Authors:** M. Zastrozhin, V. Skryabin, D. Sychev, E. Bryun

**Affiliations:** 1 Addictology, Russian Medical Academy Of Continuous Professional Education, Moscow, Russian Federation; 2 Addictology, Moscow Research and Practical Centre on Addictions, Moscow, Russian Federation

## Abstract

**Introduction:**

Mirtazapine is commonly prescribed to patients diagnosed with major depressive disorder.Some proportion of these patients do not show adequate response to treatment regimen containing mirtazapine, whereas many of them experience dose-dependent adverse drug reactions.

**Objectives:**

The objective of our study was to investigate the influence of 1846G>A polymorphism of the CYP2D6 gene on the concentration/dose indicator of mirtazapine, using findings on enzymatic activity of CYP2D6 and on CYP2D6 expression level obtained by measuring the hsa-miR-370-3p plasma levels in patients suffering from recurrent depressive disorder.

**Methods:**

Our study included 192 patients with major depressive disorder. Treatment efficacy was evaluated using the international psychometric scales. For genotyping and estimation of the microRNA plasma levels we performed the real-time polymerase chain reaction. The activity of CYP2D6 was assessed with HPLC-MS/MS method by the content of the endogenous substrate of given isoenzyme and its metabolite in urine. Therapeutic drug monitoring has been performed using HPLC-MS/MS.

**Results:**

We didn’t reveal a statistical significance for concentration/dose indicator of mirtazapine in patients with different genotypes: (GG) 0.229 [0.158; 0.468] and (GA) 0.290 [0.174; 0.526], p = 0.196). We revealed the relationship between the CYP2D6 enzymatic activity and the hsa-miR-370-3p plasma concentration: rs = -0.32, p < 0.001. At the same time, correlation analysis revealed a statistically significant relationship between the mirtazapine concentration and the hsa-miR-370-3p plasma concentration: rs = 0.31, p<0.001.

**Conclusions:**

Thus, the effect of genetic polymorphism of the CYP2D6 gene on the efficacy and safety profiles of mirtazapine was demonstrated in a group of 192 patients with recurrent depressive disorder.

**Conflict of interest:**

Authors do not have any conflict of interests.

